# Diversity in Social Communication and Support: Implications for Loneliness Among LGB Adults

**DOI:** 10.1093/geront/gnac101

**Published:** 2022-07-21

**Authors:** Natasha Peterson, Jeongeun Lee, Joseph Svec, Daniel Russell

**Affiliations:** Department of Human Development and Family Studies, Iowa State University, Ames, Iowa, USA; Department of Human Development and Family Studies, Iowa State University, Ames, Iowa, USA; Department of Human Development and Family Studies, Iowa State University, Ames, Iowa, USA; Department of Human Development and Family Studies, Iowa State University, Ames, Iowa, USA

**Keywords:** Mental health, Sexual orientation, Social network

## Abstract

**Background and Objectives:**

Current research indicates that structural and functional social network attributes influence older adults’ well-being. However, these linkages may vary by sexual orientation. This study examines how social communication diversity and support diversity are related to loneliness and differ between lesbian, gay, and bisexual (LGB) and heterosexual adults.

**Research Design and Methods:**

Using data from the American Association of Retired Persons Foundation’s Loneliness and Social Connection Survey of adults 45+ (*N* = 3,009), including 10% who identified as LGB, we derive entropy scores, which capture the extent to which network size and quality of relationships are evenly distributed. A series of linear regressions were conducted to examine sexual orientation, social network indicators, and their interactions in predicting loneliness.

**Results:**

We found a positive association between social communication diversity and loneliness. This finding was qualified by the interaction with sexual orientation. In addition, we found a negative association between support diversity and loneliness, an effect that was stronger for LGB participants than for heterosexual participants. The effect of support diversity on loneliness was much stronger for LGB adults than heterosexual adults.

**Discussion and Implications:**

While LGB adults tend to score higher on the loneliness scale overall, the findings suggest that communication and support diversity have uniquely different patterns of associations for sexual minority groups. This study highlights the importance of considering multiple dimensions of social networks and has implications for addressing loneliness for heterosexual and LGB adults.

Social connection, or the experience of feeling close or connected to others, is vital for health and well-being (Hawkley & Cacioppo, 2010). Based on a national survey conducted by the American Association of Retired Persons (AARP), more than one third (35%) of adults 45 and older in the United States are lonely (Anderson & Thayer, 2018). Given the profound mental and physical health consequences of loneliness, such as increased rates of depression and premature death (Hawkley & Cacioppo, 2010; Krendl & Perry, 2021; Park et al., 2020), social engagement among network members warrants attention. Leading predictors of loneliness include the size and diversity of one’s social network and being physically isolated (Anderson & Thayer, 2018). As social technology has gained popularity and has both positive and negative impacts on social networks, we examine whether and the extent to which the frequency and type of communication with different network members matter for loneliness. Additionally, research shows that vulnerable groups, such as lesbian, gay, and bisexual (LGB) adults, are at a greater risk for loneliness (Anderson & Thayer, 2018). Thus, this study compares middle-aged and older LGB and heterosexual adults’ social networks, their communication methods, and loneliness levels.

The importance of social networks on the mental health and well-being of adults generally is well documented (Domènech-Abella et al., 2017; Li & Zhang, 2015). However, such findings are limited by social groups, and the lack of inclusion or consideration of differential experiences can have profound implications for the extant literature. For example, middle-aged and older LGB adults tend to have unique patterns in their social networks—often including “families of choice” or more friend-centered relationships—due to adverse experiences of discrimination and exclusion across their life course (Fredriksen-Goldsen et al., 2013; Hsieh & Liu, 2021; Hughes et al., 2021; Kim et al., 2017; Reczek, 2020). Moreover, LGB adults are more likely to live alone and to be single and childless (Fredriksen-Goldsen et al., 2013). Consequentially, their distinct network size and levels of support may put older LGB adults at greater risk for social isolation and loneliness than their heterosexual counterparts (Erosheva et al., 2016).

The advent of social technology has altered our communication methods. Communication via social media, e-mail, text messaging, and video chatting has led to the diversification of our social networks and frequencies of communication with social network members (Teo et al., 2015; Yuan et al., 2016; Zhang et al., 2021). Researchers have found that diverse social networks embedded with technology are associated with increased social capital and well-being (Cohen et al., 2000; Cohen & Janicki-Deverts, 2009, Licoppe & Smoreda, 2005). Such studies often rely on simple counts of social network members in quantifying the impact on social networks. However, examining relative communication across multiple social partners may provide useful information regarding the benefit of diversified social communication and support. In this research, we aim to expand existing knowledge by using a nationally representative sample to examine the implication of social communication diversity and support diversity on loneliness, comparing this effect by sexual orientation.

## Minority Stress Model

The minority stress model suggests that minority groups, such as sexual minorities, experience stress processes, including stressful events like discrimination and prejudice, concealment of one’s LGB identity, internalized homophobia, expectations of rejection, and ameliorative factors (e.g., ingroup cohesiveness; Hatzenbuehler, 2009; Kuyper & Fokkema, 2010; Meyer, 2003). Hence, minority stress is associated with higher risks for mental and physical health outcomes (Hsieh et al., 2021; Meyer, 2003). Given the uniqueness of their social convoys (i.e., smaller, friend-centered, childless) or social embeddedness due to experiences with discrimination related to their sexual orientation, LGB adults may be prone to greater loneliness (Fredriksen-Goldsen et al., 2011; Kuyper & Fokkema, 2010; Lam & Campbell, 2022). Moreover, the fear of disclosure of their sexual orientation may limit social relations, in turn, developing smaller and less diverse contacts (Erosheva et al., 2016). Social network size and satisfactory social support may serve as protective factors (Erosheva et al., 2016; Fredriksen-Goldsen et al., 2013). Using the minority stress model as a framework, we can understand how sexual minorities are situated in distinct social networks, including differential aspects of communication and support, and if those social networks are related to loneliness in mid to late adulthood.

## Theoretical and Empirical Literature on Social Networks

The convoy model of social relations offers a framework for conceptualizing the ways in which social networks or “convoys” influence positive or negative mental health outcomes, such as loneliness, throughout the life course (Antonucci, 2001; Antonucci et al., 2014). The model asserts that social relations are multidimensional, differing by structure, function and support, and quality and satisfaction (Antonucci, 2001). Structure refers to the network size, composition (family or friends), contact mode (text or phone) and frequency, and geographic proximity (Antonucci et al., 2014). Social support refers to the functional exchange of support. Lastly, satisfaction and quality refer to the individual’s evaluation of that exchange (e.g., positive or negative). These networks can change, gaining and losing members based on situational (e.g., norms) or personal contexts (e.g., age, gender). LGB adults have particular life experiences that influence who is in their network and their patterns of social support over time (Fuller et al., 2020).

Literature provides differential evidence on the structural and functional aspects of social networks (Fiori et al., 2006; Litwin & Shiovitz-Ezra, 2006). Support for the structural aspect of the social network is found in physical health outcomes, as a broader array of network members can promote physical and cognitive functioning (Litwin & Shaul, 2019). In addition, having social networks that are large and composed of frequent contact has also been associated with lower levels of social isolation, loneliness, and depression among older adults (Cacioppo et al., 2010; Domènech-Abella et al., 2017; Goodman et al., 2021). This is consistent with the strength-of-weak-ties proposal (Granovetter, 1983)—diverse relationship types ultimately serve as resources to access many opportunities (Erosheva et al., 2016; Miritello et al., 2013). On the other hand, other studies have found that functional aspects of social networks, which are captured by the supportive members in the social network, can benefit the emotional well-being of older adults more so than structural aspects of networks (Carstensen, 2021; Dupertuis et al., 2001).

## Social Communication Diversity

One way that social convoys influence the lives of older adults is through social communication. Previously, studies have not clearly examined whether or the extent to which *social communication diversity*, defined as the extent to which communication is evenly distributed among existing social ties, is beneficial for the well-being of older adults. Some studies, for example, find that increases in social communication methods, such as using information and communication technologies with many social partners, can alleviate social isolation and feelings of loneliness (Chopik, 2016; Khosravi et al., 2016). Most studies focus on adding new types of social communication to existing communication methods with various types of social network members. Using a communication diversity method, Alshamsi et al. (2016) found that social network communication diversity correlates with both positive and negative states. Although this study highlights the effect of social communication diversity on emotional affect, it is limited to two types of communication methods, including face-to-face or mobile phone communication. Because most middle-aged and older adults are now using various types of communication methods (e.g., text messaging, e-mail) in addition to in-person and telephone conversations to communicate with their social network (Fuller et al., 2020; Zhang et al., 2021), a more comprehensive assessment would provide a clearer picture of the role of social communication diversity and its effect on loneliness. Thus, we aim to capture social communication diversity, which can assess the structural properties of dynamic social networks of individuals with diverse methods of communication.

## Support Diversity

While studies have examined the differential predictive value of structural and functional aspects of social relationships (van den Brink et al., 2018), few examine whether the combination of these two factors contributes to loneliness. In this regard, we define *support diversity* as the interaction between the number of supportive people and social communication diversity. As not all social connections and communications positively affect people’s lives (Rook, 2015), having frequent contact with diverse social network members may create more tensions and conflicts rather than reduce loneliness. As individuals’ subjective recognition of support (i.e., perceived support) has been associated with reduced loneliness (Chen & Feeley, 2014), a more complete understanding might be achieved by considering the ways in which structural and functional characteristics play different roles in relieving loneliness. Given the configuration of social networks that LGB adults have (Erosheva et al., 2016; Kim et al., 2017), it is plausible that these different aspects of social relationships, as well as the combination of the two, are differentially associated with loneliness.

As noted above with the Minority Stress Theory and empirical findings, support diversity may have different implications for LGB adults given their unique social networks (Erosheva et al., 2016; Kim et al., 2017). Given that a small amount of evidence has shown that older adults’ social capital is generally associated with lower loneliness, we considered how LGB adults might lack diverse social networks and support, which can culminate in elevated loneliness levels. Therefore, we examine whether the association between the structural and functional characteristics of social networks and the loneliness of LGB adults may be related.

## Aim of the Study

Studies largely show that meaningful social networks positively affect the mental health of older adults (Domènech-Abella et al., 2017; Li & Zhang, 2015). More specifically, having a wider social network has implications for the well-being and health of LGB adults (Fredriksen-Goldsen et al., 2014; Hsieh et al., 2021). However, in the current literature, the breadth and depth of social ties have often been examined separately, which may overlook the nuanced outcomes attributed to their combined effects on loneliness. To our knowledge, although the salient role of the social network and support on loneliness among LGB adults may differ, the comparative effect has not been examined. Such a comparison will enhance our understanding of the impact of diversified modes of communication with one’s social network. This research fills a gap in the literature regarding how these connections vary for marginalized groups.

Based on the extant literature, we expect that having broader social communication and support diversity corresponds with reduced levels of loneliness. Because LGB midlife and older adults have a smaller social circle to rely on, they may be more vulnerable to loneliness (Kim et al., 2017). Drawing on empirical and theoretical literature on social relationships as well as the minority stress model, we test four hypotheses regarding differences in heterosexual and LGB adults’ associations with loneliness.

Hypothesis 1: Increased levels of social communication diversity correspond with lower levels of loneliness.

Hypothesis 1a: The association between social communication diversity and loneliness differs significantly by sexual orientation. In particular, we expect that social communication diversity will have a stronger effect on loneliness among LGB adults.

Hypothesis 2: Increased levels of support diversity correspond with lower levels of loneliness.

Hypothesis 2a: The association between support diversity and loneliness differs significantly by sexual orientation. Specifically, we expect that the effect of support diversity on loneliness will be stronger for LGB adults.

## Method

### Design and Participants

Data from the 2018 Loneliness and Social Connections Survey conducted by the AARP Foundation were utilized for this study (Anderson & Thayer, 2018). A total sample of 6,343 adults aged 45 aged and older from the continental United States was recruited to complete the main survey from the KnowledgePanel, a probability-based web panel designed to be representative of the United States. Recruitment was based on an Address-Based Sampling methodology. To be eligible for participation, respondents had to be at least 45 years of age and speak English or Spanish. A total of 3,223 completed the main survey: 2,905 who identified as heterosexual and 318 who identified as LGB. Study-specific poststratification weights were applied in the analyses.

### Measures

#### Loneliness

Loneliness is measured using the UCLA Loneliness Scale (Russell, 1996). This scale includes 20 questions that are responded to on a 4-point scale, ranging from 1 (*never*) to 4 (*always*). Example items include, “How often do you feel that you lack companionship?” and “How often do you feel that you are outgoing and friendly?” Scores ranged from 20 to 77 (*M* = 39.61, *SD* = 11.16, α = 0.94).

### Social Network and Communication

Literature on social networks has emphasized the structural dimensions of social networks, including the frequency of the contacts and dispersion of social network members (Berkman & Syme, 1979; Seeman et al., 1987). First, to assess the structural characteristics of the social network, we evaluated social communication diversity using entropy scores, which capture both the size of the network and the communication modalities employed with network members. Second, given the importance of the quality of social network relationships (Dahlberg et al., 2021; Lee et al., 2017), we assessed the number of supportive social network members. Finally, we assessed support diversity by multiplying both supportive social network members and social communication diversity.

#### Social communication diversity

To construct the measure of social diversity, we adapted Shannon’s entropy method, which has been used to quantify emotional diversity, activity diversity, and social diversity (Shannon, 1948). We utilized Shannon’s entropy scores to quantify dispersed communication channels and network members.

**Figure 1.**
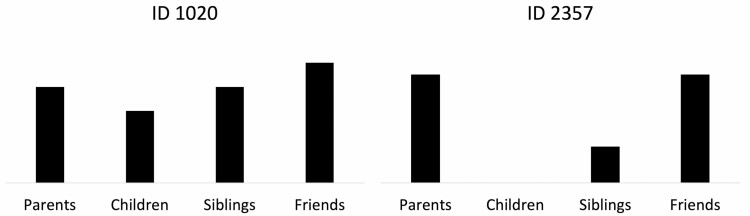
Examples of social communication diversity.

To assess the size and the frequency of contact, we estimated social communication diversity scores. Participants were asked how often they contacted the following types of people: parents, children, siblings, and friends with seven different types of communication methods [in person, e-mail, telephone, letters or postcards, text messaging, online messaging or video messaging chat (e.g., Skype, Facetime)], and social networking sites (e.g., Facebook, Twitter, Instagram). Overall, 28 social communication items across four types of relationships are asked in total. We then collapsed all types of communication methods to estimate the frequency of the communication across the relationships (see Figure 1). These two indicators (social communication frequency and social network members) were used to calculate the entropy scores described below.

Second, we calculated Shannon’s entropy scores to represent social communication dispersion across relationships within participants (Ram et al., 2012): social communication diversity = $-\left(\frac{1}{\ln(m)}\right)\sum_{j=1}^{m}p_{ij}\ln p_{ij}$, where the entropy score for a person is a function of the proportion of (*p*_*ij*_) of the frequency of social communication indicators (social communication indicators 1 to *m*). The entropy scores were then scaled to range from 0 (no social communication indicator diversity) to 1 (i.e., similar levels of social communication indicators across relationships). Figure 1 displays two individuals with different social communication diversity. As can be seen, individual (ID 1020) engages in frequent and fairly even communication with several social network members, whereas individual (ID 2357) engages in frequent communication with limited social network members. There is greater variability across groups for individual (ID 2357). In this example, ID 2357 has higher social communication diversity than ID 1020.

#### Number of supportive people

The number of supportive network members was also asked. First, the question asked how many people have been supportive of participants during the past year. After participants provided the initial number, the follow-up prompt asked who they were. Eight different types of relationships (friends, spouse or partners, children, parents, other relatives, neighbors, coworkers, and other relationships). The first number and the combined number from the second inquiry matched. Thus, we assigned one point for each relationship. The mean score was 2.89 (*SD* = 1.54).

#### Support diversity

As noted above, we also measure support diversity via communication by creating an interaction term computed by multiplying supportive network members and social communication diversity score (entropy score). This supportive communication diversity score indicates the extent to which individuals utilize social communication methods with supportive members in their social networks. Given the way support diversity is constructed, a higher level of support diversity would indicate support from the social network members is communicated through diverse social communication methods.

#### Background characteristics

Participants were also asked to provide sociodemographic information: age (in years), gender (men = 1, women = 0), sexual orientation (1 = LGB, 0 = heterosexual), race/ethnicity (1 = non-Hispanic White, 0 = other), education (1 = high school or lower, 2 = some college, 3 = Bachelor’s degree of higher), number of household members, relationship status (1 = partnered, 0 = single), number of mental health conditions (sum score), number of physical health conditions (sum score), and household income (1 = low, 2 = moderate, 3 = high).

### Analysis Plan

All analyses were conducted in Stata (15.1, StataCorp LLC, College Station, TX). First, we estimated correlations between outcome variables and covariates. Because covariates that are not associated with the dependent variable may generate spurious associations between variables (Rovine et al., 1988), we eliminated the variables that were not significantly correlated with outcome variables (i.e., race and number of household members). Based on the correlations with outcome variables, all models controlled for several individual characteristics (i.e., age, gender, education, relationship status, mental health, physical health, and household income). The following interaction terms (multiplying variables together) were created for analyses: social communication diversity × sexual orientation, social communication diversity × number of support network members (we label this as support diversity), and support diversity × sexual orientation.

To test the hypotheses, we conducted a series of linear regression models with listwise deletion of missing data (see Table 2). All four models had loneliness as the dependent variable and included covariates. More specifically, to test Hypothesis 1, the first model included sexual orientation, social communication diversity, and the number of supportive network members. Keeping the same predictors from Model 1, the interaction term between sexual orientation and social communication diversity was added to Model 2 to test Hypothesis 1a. To examine Hypothesis 2, support diversity, or the interaction term between social communication and the number of supportive members, was the only predictor in Model 3 besides covariates. Lastly, Model 4 included support diversity and the interaction between support diversity and sexual orientation to assess Hypothesis 2a.

## Results

The descriptive statistics of the total sample, the LGB group, and the heterosexual group are shown in Table 1. Among the 3,009 participants in the final analytic sample, the mean age of the respondents was 62.38 (*SD* = 10.11), approximately half (49.68%) were men, and a majority (67.83%) were in a relationship. Educational attainment was fairly distributed across the three groups (31%–36%). Out of a maximum of nine, the average number of supportive people in participants’ lives was approximately three. The social communication diversity score had a mean of 0.38 (*SD* = 0.19). Overall, the average loneliness score is 39.61 (*SD* = 11.16) out of a range from 20 to 77. It should be noted that there are significant differences between the LGB and heterosexual groups on all of the variables included in the analyses.

**Table 1.** Descriptive Statistics (*N* = 3,009)

	Min	Max	Total (*N* = 3,009)	Heterosexual (*n* = 2,705)	LGB (*n* = 304)	*t* Test
Variables			Mean/%	Mean/%	Mean/%	
Loneliness score	20	77	39.61	39.11	44.07	−7.42***
Social communication diversity score	0	0.71	0.38	0.39	0.36	2.09*
Number of supportive people	0	9	2.89	2.93	2.50	4.63***
Support diversity score	0	5.70	1.14	1.16	0.95	3.94***
Age	45	93	62.38	62.68	59.67	4.94***
Men	0	1	49.68%	47.6%	64.5%	−5.71***
Education	0	3	2.03	2.01	2.20	−3.78***
High school or lower (ref)				33.97%	24.01%	
Some college				30.94%	31.91%	
Bachelor’s degree or higher				35.08%	44.08%	
In a relationship	0	1	67.83%	70.20%	46.71%	8.41***
Mental health	0	2	0.30	0.30	0.51	−6.18***
Physical health	0	8	1.33	1.31	1.50	−2.49*
Household income	1	3	1.92	1.93	1.80	2.96**
Low				31.83%	38.49%	
Moderate				42.99%	43.09%	
High				25.18%	18.42%	

*Notes*: LGB = lesbian, gay, and bisexual.

***p < .001. **p < .01. *p < .05.

We conducted several linear regression analyses to test our hypotheses (see Table 2). Across models, loneliness levels were higher by 1.63 to 4.28 units for LGB adults compared to heterosexuals, *p* < .001.

**Table 2.** Linear Regression Analyses Examining Sexual Orientation and Social Network Indicators on Loneliness Levels

Variables	Model 1 *B* (*SE*)	Model 2 *B* (*SE*)	Model 3 *B* (*SE*)	Model 4 *B* (*SE*)
Identifies as LGB	1.63** (0.61)	4.28*** (1.29)	1.74** (0.63)	3.17*** (0.96)
Social communication diversity	2.25* (0.95)	2.96** (0.99)		
Number of supportive people	−2.06*** (0.12)	−2.06*** (0.12)		
Interaction: LGB × Social communication diversity		−7.27* (3.11)		
Support diversity			−2.13*** (0.21)	−2.01*** (0.22)
Interaction: LGB × Support diversity				−1.47* (0.74)
Age	−0.21*** (0.02)	−0.21*** (0.02)	−0.22*** (0.02)	−0.22*** (0.02)
Men	0.32 (0.37)	0.32 (0.37)	0.77* (0.38)	0.78* (0.38)
Education (ref: High school or lower)				
Some college	0.33 (0.45)	0.37 (0.45)	0.24 (0.47)	0.25 (0.47)
Bachelors or higher	0.98* (0.48)	0.98* (0.48)	0.86 (0.49)	0.87 (0.49)
In a relationship	−2.42*** (0.42)	−2.41*** (0.42)	−2.75*** (0.43)	−2.74*** (0.43)
Mental health	3.58*** (0.31)	3.59*** (0.31)	3.80*** (0.32)	3.80*** (0.32)
Physical health	0.71*** (0.15)	0.71*** (0.15)	0.70*** (0.15)	0.70*** (0.15)
Household income (ref: Low)				
Moderate	−1.04* (0.45)	−1.00* (0.45)	−1.36** (0.46)	−1.34** (0.46)
High	−2.76*** (0.57)	−2.72*** (0.57)	−3.19*** (0.58)	−3.17*** (0.58)
Constant	57.79*** (1.39)	57.46*** (1.40)	56.28*** (1.35)	56.08*** (1.35)
Observations	3,009	3,009	3,009	3,009
*R* ^2^	0.24	0.24	0.19	0.19

*Notes*: LGB = lesbian, gay, and bisexual; *SE* = standard error. The outcome variable is the loneliness score. *B* is the unstandardized regression coefficient.

***p < .001. **p < .01. *p < .05.

In Table 2, Model 1 shows the main effects of sexual orientation, supportive network size, and social communication diversity on loneliness. This model predicted 24% of the variance in loneliness level, *R*^2^ = 0.24, *F*(12, 2996) = 77.22, *p* < .001. There was a positive association between social communication diversity and loneliness (*B* = 2.25, *p* < .01); hence, greater social communication diversity predicted greater loneliness (H1 not supported).

**Figure 2.**
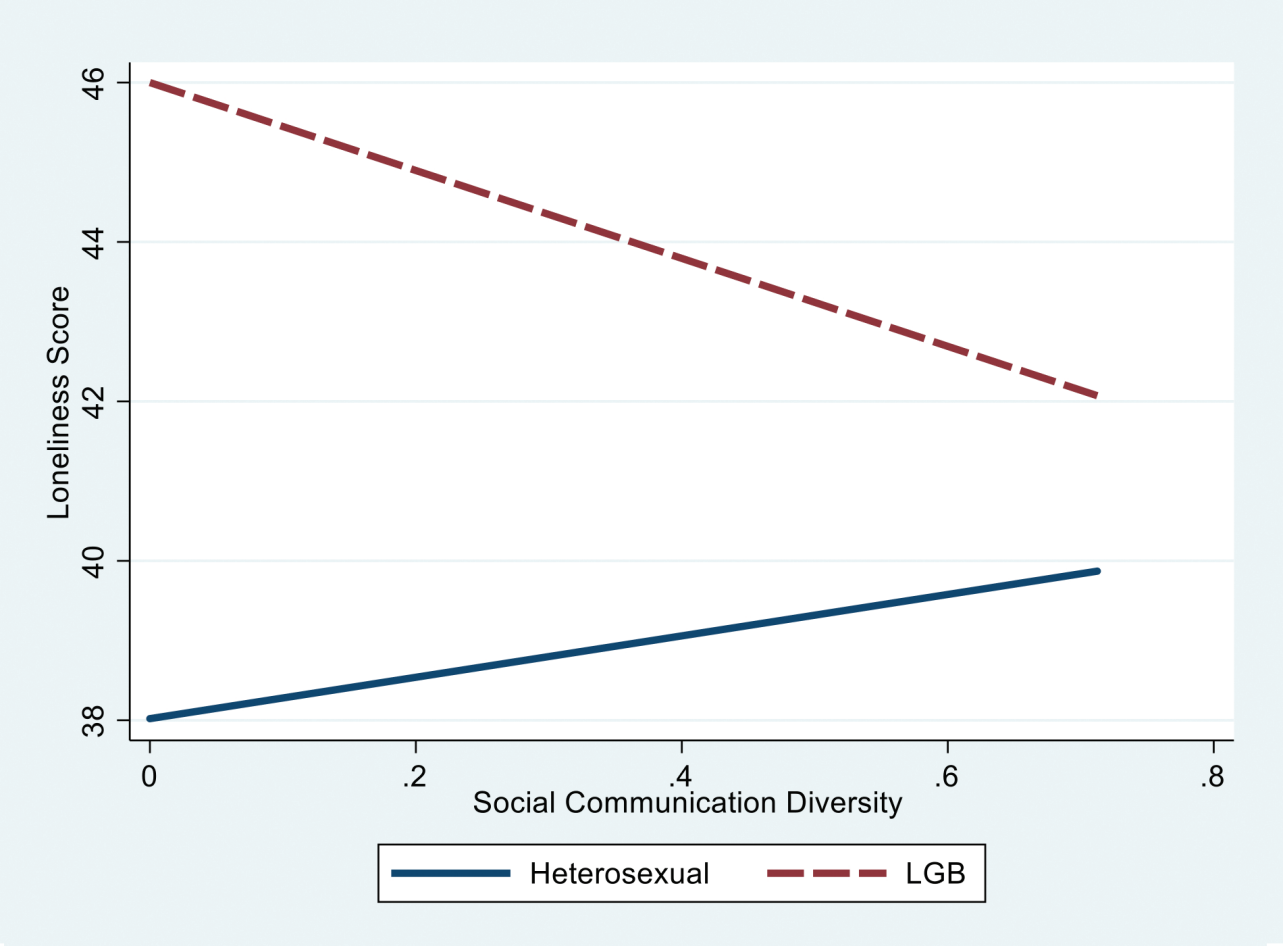
The interaction between social communication diversity and sexual orientation on loneliness levels. LGB = lesbian, gay, and bisexual.

To test whether the association between social communication diversity and loneliness functioned similarly for LGB and heterosexual individuals (H1a), we added the interaction term between social communication diversity and sexual orientation in Model 2 of Table 2. These predictors explained 24% of the variance, *R*^2^ = 0.24, *F*(13, 2995) = 71.81, *p* < .001. The interaction was significant and negative (*B* = 7.27, *p* < .05). As seen in Figure 2, we found the opposite pattern. For heterosexual adults, higher social communication diversity was positively linked with their feelings of loneliness. In contrast, for LGB adults, higher social communication diversity was associated with lower levels of loneliness.

To examine our H2, we included support diversity in Model 3 of Table 2. Supplementary Figure 1 shows how this measure was used. Support diversity, which takes into account the number of supportive network members and social communication diversity, had a negative association with loneliness (*B* = −2.13, *p* < .01). As expected, greater support diversity was associated with lower levels of loneliness. This model predicted 19% of the variance in loneliness levels, *R*^2^ = 0.19, *F*(11, 2997) = 63.15, *p* < .001.

**Figure 3.**
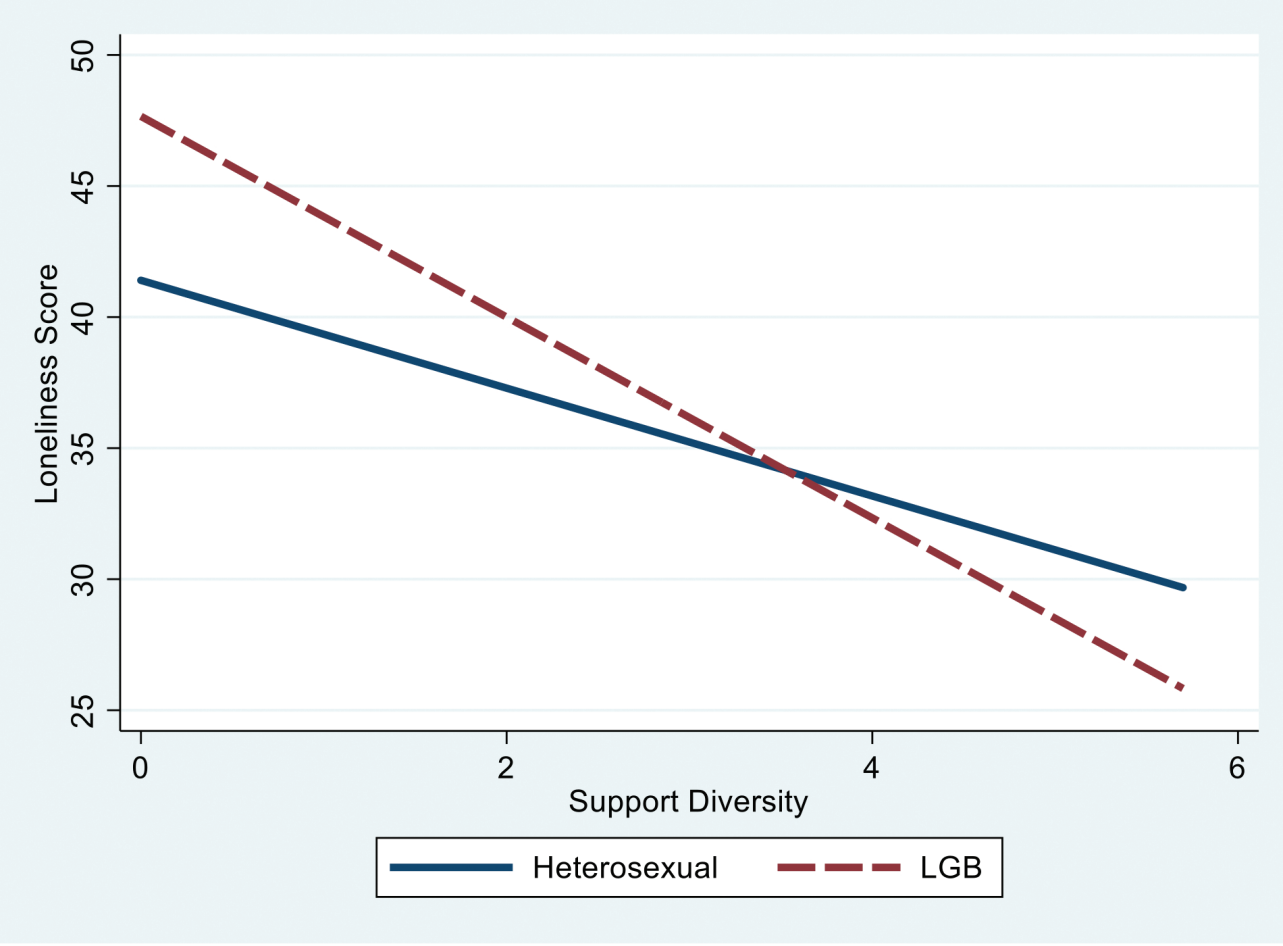
The interaction between support diversity and sexual orientation on loneliness levels. LGB = lesbian, gay, and bisexual.

To examine H2a, we included the interaction term between support diversity and sexual orientation (see Model 4 in Table 2). Support diversity, the interaction between support diversity and sexual orientation, and the covariates predicted 19% of the variance in loneliness levels (*R*^2^ = 0.19, *F*(12, 2996) = 58.27, *p* < .001). The interaction term was significant and negative (*B* = −1.47, *p* < .05). As depicted in Figure 3, LGB adults with lower support diversity reported greater loneliness than heterosexual adults who also had lower support diversity. When support diversity increases, the levels of loneliness among heterosexual adults are higher than those of LGB adults. Support diversity seems to matter more for LGB adults than heterosexual adults. Overall, our findings support our last hypothesis that social communication and support predicting loneliness would depend on sexual orientation.

All four models in Table 2 included control factors that were associated with loneliness. Age was negatively associated with loneliness in all models, such that increased years in age predicted lower loneliness, *p* < .001. Similarly, relationship status along with the number of mental health and physical health conditions consistently contributed to loneliness levels, *p* < .001. Being in a relationship reduced loneliness, whereas having more mental and physical health conditions increased loneliness. Gender was a significant predictor of loneliness levels in only the last two models with support diversity and the interaction between support diversity and sexual orientation, *p* < .05; men were more likely to be lonely than women. Conversely, having a Bachelor’s degree or higher resulted in higher loneliness than the reference group (high school or lower) for only the first two models, *p* < .05. Compared to those with low income, those with moderate and high income had lower loneliness across all models, *p* < .05.

Post hoc analyses were conducted to assess differences in communication types: face-to-face and remote communication (e.g., e-mail, social media, text, call, video chat, letters). Heterosexuals were more likely to meet in person (*M* = 12.03, *SD* = 0.07) than LGB adults (*M *= 10.13, *SD* = 0.22), *t*(3,007) = 8.29, *p < *.001. The frequency of meeting in person was added to the regression analysis predicting loneliness in Supplementary Table 1. The frequency of meeting in person was negatively related to loneliness (*B* = −0.54, *p < *.001). However, the interaction between sexual orientation and the frequency of meeting in person was nonsignificant. Heterosexuals were also more likely to engage in remote communication (*M *= 50.85, *SD = *0.35) than LGB participants (*M* = 44.84, *SD* = 1.02), *t*(3,007) = 5.43, *p < *.001. However, when taking a closer look at who participants interact with, LGB participants (*M *= 22.69, *SD* = 0.35) were more likely to communicate with friends than heterosexual participants (*M *= 20.94, *SD* = 0.12), *t*(3,110) = −4.74, *p < *.001, whereas LGB adults (*M *= 8.63, *SD* = 0.56) were less likely to communicate with children than their counterparts (*M *= 16.34, *SD = *0.18), *t*(3,007) = 13.71, *p < *.001. There were no significant differences between groups on communication with siblings or parents.

## Discussion

In this study, we examine the differential association between social communication diversity, support diversity, and loneliness among middle-aged and older LGB adults to heterosexual adults. While LGB adults tend to score higher on the loneliness scale overall, the findings suggest that communication and support network diversity have different patterns of associations between LGB and heterosexual individuals. The findings have numerous implications for both pragmatic and functional considerations of addressing loneliness issues as well as theoretical understandings of social support systems more generally.

Ultimately, the data show a positive association between social communication diversity and loneliness, contradicting our initial hypothesis that greater diversity corresponds with lower levels of loneliness. These unexpected findings may constitute a manifest paradox of communication and mental health. For example, a greater number of ties is at times shown to correspond with higher levels of stress and is depicted as emotionally draining, given the higher exertion of physical and mental activity to maintain those ties (Kawachi & Berkman, 2001; Miritello et al., 2013). This finding is in line with the Socioemotional Selectivity Theory, which posits that older adults narrow their social networks as they age to allocate more emotional resources to fewer relationships (Carstensen, 2021). While social connections and communication often go hand-in-hand with feelings of solidarity and closeness, heightened levels of communication may also stretch individuals beyond comfort (Roberts & Dunbar, 2011).

In contrast to social communication, the association between support diversity and loneliness is negative. This indicates that the interaction between social communication diversity and support network size corresponds with lower levels of loneliness as expected (H2). This further complicates the positive association between social communication diversity and loneliness as larger and more diverse social networks are linked with lower levels of loneliness. However, we posit that the results show the importance of understanding relationship quality as a multidimensional concept. A highly diverse social network, but one lacking in deeper and supportive members, may not ameliorate feelings of overall loneliness (Fuller et al., 2020). With the inclusion of broader sentiments of feeling well supported in the assessment of social communication diversity, these data suggest that relationship quality is not an “either-or” proposition. Rather, the quality of networks, which incorporate both the extent of support and the diversity of that support, may more accurately predict feelings of inclusion and loneliness (Hawkley et al., 2008). Such conclusions are well alluded to in the literature in which scholars suggest that relationship quality may matter more than relationship quantity (Hawkley et al., 2008; Pinquart and Sörensen, 2003; Schmidt et al., 2021).

A primary motivation for this study was to examine the differential associations of social network compositions and loneliness by sexual orientation. The models support both hypothesized interactions for social communication diversity (H1a) and support diversity (H2a).

Interestingly, our findings indicated a significant interaction between social communication diversity and sexual orientation, such that higher social communication diversity was associated with greater loneliness only among heterosexuals. The effect was the opposite for LGB adults: higher social communication diversity was linked with lower levels of loneliness. For heterosexuals, having equal levels of contact with various social ties may be an attempt to reduce loneliness or be connected. Longitudinal and sequential design that directly tests social communication diversity and loneliness in a similar time frame will allow us to test this relationship. Considering that LGB adults are more likely to live alone, less likely to be in a relationship, and less likely to have children (Fredriksen-Goldsen et al., 2011), they tend to have less contact with others, giving them fewer opportunities to form and maintain relationships across their life span. According to the minority stress model, less contact with network members may also be a result of trying to conceal their sexual identity (Meyer, 2003). Instead, they may rely more heavily on social technology (e.g., text, Facetime, social media) to connect with their “families of choice” (Escobar-Viera et al., 2018; Mock et al., 2020). We found this to be true in our post hoc analyses. LGB adults were more likely than heterosexuals to communicate with friends. Overall, this finding may point to high social communication diversity as a protective factor for LGB adults.

We found support for our last hypothesis that support diversity would depend on sexual orientation. Relative to heterosexuals, support diversity seems to matter more for LGB adults. LGB adults were lonelier with less support diversity and had a steeper negative slope with more support diversity. Consistent with the social convoy model (Antonucci et al., 2014), social relations have multiple, distinct elements (i.e., structure, function, quality). LGB adults may rely more heavily on their social networks, particularly meaningful friendships, than heterosexuals for their mental health (Tester & Wright, 2017). An empirical study conducted by Fredriksen-Goldsen et al. (2013) found related findings—increased social support and social network size were attenuating factors against poor health outcomes for LGB older adults.

Higher loneliness among LGB versus heterosexuals is consistent with previous studies and the minority stress model (Fredriksen-Goldsen et al., 2011; Hughes, 2016; Kim et al., 2017; Kuyper & Fokkema, 2010). More fragile networks may stem from disconnected biological families and not finding similar people to connect with (e.g., older LGB people). Also aligning with prior literature, loneliness was partially explained by relationship status, age, gender, mental health, physical health, education status, and income. For example, some studies indicate that being in a relationship is linked to lower loneliness for both heterosexuals and sexual minorities (Kim & Fredriksen-Goldsen, 2016; Kuyper & Fokkema, 2010; Pinquart, 2003). Contrary to popular belief, older adults are not more lonely than young adults (Beam & Kim, 2020). The literature suggests that loneliness is nonlinearly related to age—increasing in adolescence and late adulthood after age 75 (Hawkley et al., 2019).

The results have implications for scholarship and interventions. The measurement of social communication diversity should be considered in understanding the patterns and trends of older age loneliness. As the methods of communication continue to adapt to new environmental and technological conditions, the structural opportunities for individuals to interact with one another have direct consequences for mental and physical health. Incorporating knowledge of *who* and *how* people interact with their social networks can offer new insights and avenues for addressing the growing issue of loneliness and isolation. This research adds to the growing literature on loneliness by taking a multidimensional approach to measuring and conceptualizing social network composition.

The core of this research is the differing associations of social communication and support diversity for heterosexual and LGB adults. The finding that social communication diversity, while positively associated with loneliness for heterosexual adults and negatively associated for LGB adults, has significance for the dynamics of loneliness more generally. We suggest that the differing associations for marginalized groups, who often exist in constrained social convoys, may depict a nuanced framework of social network implications. The diversity of social networks and communication may also reflect the social opportunities and ascribed meanings behind social classes. As suggested in the minority stress perspective (Meyer, 2003), coping and health behaviors are not purely individualized navigation of generic psychological processes but are also framed by the social positions in which one resides. Thus, relevant interventions would do well to consider both geographic and social positionality. From a theoretical perspective, these results further highlight the importance of unpacking the intersectional configurations of individual profiles and social conditions.

### Limitations of the Study

Our measure of social communication diversity summarized the dispersion of activity across social communication methods without drilling into the specific types of social communication methods. Given how the entropy scores were derived, low social communication diversity does not necessarily indicate a less cohesive relationship with social network members. As our results showed, some participants may prefer and maintain frequent contact with fewer social network members.

Moreover, the social convoy model, which incorporates structure, function, and quality, is used as one of the conceptual models guiding the study (Antonucci et al., 2014). However, the data do not have appropriate measures of satisfaction or quality of support. It is unknown whether interactions among networks were positive or negative. Albeit, the number of supportive people was included in the analyses, which does capture some aspects of network quality. In addition, loneliness, by definition, is the dissatisfaction with one’s social relationships (Hawkley & Cacioppo, 2010). Nonetheless, future studies using the social convoy model as a guiding framework should thoroughly inspect all three aspects.

In addition, the nature of the selected sample may have limited the identification of relations between social communication diversity and loneliness. Most participants were healthy and independently residing in the community. Future research should look for ways to include less healthy and the oldest adults to see whether current findings are replicated in a sample with less capability. Also, this study draws on data before the coronavirus disease 2019 (COVID-19) pandemic. Thus, participants’ familiarity and wide use of Zoom had not yet occurred. Given how technology usage has changed, researchers may want to replicate similar studies using post-COVID era data. Furthermore, the current study is a cross-sectional study, which does not allow us to discern the causal direction of effects. Changes in social communication diversity may lead to loneliness, but the reverse may be true, such that loneliness leads to changes in social communication diversity. It is plausible that loneliness could lead to the underreporting of social ties.

It is also important to note how demographic patterns of structural advantage or disadvantage (e.g., race, gender, age, sexual orientation, social class, geographic location) can affect social networks (Antonucci et al., 2014; Cronin & King, 2014). Not all participants have the same access to diverse social networks and the resources and support that emerge as a result (Cronin & King, 2014; Kittle et al., 2022). It is also possible that participants with limited resources may have less access to heterogeneous networks and fewer means to communicate with their social network members (Erosheva et al., 2016). For instance, an LGB individual living in a predominantly heterosexual culture in a rural area may not have access to an immediate LGB community, so they may turn to social technology as an important source of social capital.

## Conclusion

Despite these limitations, this study is one of few to consider multiple dimensions of social networks among LGB and heterosexual adults. Our findings provide valuable information about theoretically and empirically relevant social networks as different combinations of communication methods across relationships. Our findings also have practical implications for LGB adults’ mental health; understanding whole social network dimensions, not just specific aspects of social networks, might be critical to promoting better health and well-being.

## Supplementary Material

gnac101_suppl_Supplementary_Material

## Data Availability

The data used for this study were the “Loneliness and Social Connections: A National Survey of Adults 45 and Older (2018).” Researchers can request access to this data set on the AARP website: https://www.aarp.org/research/data-tools/datasets/.
