# Supplementary material for: Diversity in Social Communication and Support: Implications for Loneliness Among LGB Adults
Source: Gerontologist. 2022 Jul 21;63(2):361–72. doi: 10.1093/geront/gnac101 (PMC9960016; doi:10.1093/geront/gnac101)
Supplement: gnac101_suppl_Supplementary_Material [file gnac101_suppl_supplementary_material.docx]

**Supplementary Figure 1**

*Support Diversity: The interaction between social communication diversity and the number of supportive network members on loneliness levels*

*Note*: SCD = Social Communication Diversity.

**Supplementary Table 1**

*Linear regression analysis examining sexual orientation and frequency of meeting in person on loneliness levels*

| Variable | Loneliness Score  *B*(*SE*) |
| --- | --- |
| Identifies as LGB | 2.61 |
|  | (1.73) |
| Frequency of Meeting In-Person | -0.54^***^ |
|  | (0.05) |
| Interaction: LGB X Frequency of Meeting In-Person | -0.15 |
|  | (0.16) |
| Age | -0.26^***^ |
|  | (0.02) |
| Men | 0.67^*^ |
|  | (0.38) |
| Education (Ref: High school of lower) |  |
| Some College | 0.04 |
|  | (0.47) |
| Bachelors or Higher | 0.24 |
|  | (0.49) |
| In a relationship | -2.88^***^ |
|  | (0.43) |
| Mental health | 3.65^***^ |
|  | (0.32) |
| Physical health | 0.70^***^ |
|  | (0.15) |
| Household Income (Ref: Low) |  |
| Moderate | -1.39^***^ |
|  | (0.46) |
| High | -3.39^***^ |
|  | (0.58) |
| Constant | 63.35^***^ |
|  | (1.65) |
| Observations | 3,009 |
| R-squared | .19 |

*Note.* ^***^ *p* < 0.001, ^**^ *p* < 0.01, ^*^ *p* < 0.05.
